# Tertiary Lymphoid Structures in Human Melanoma: Molecular Mechanisms and Therapeutic Opportunities

**DOI:** 10.3390/cells14171378

**Published:** 2025-09-04

**Authors:** Gelare Ghajar-Rahimi, Ishika Patel, Nabiha Yusuf

**Affiliations:** Department of Dermatology, Heersink School of Medicine, University of Alabama at Birmingham, Birmingham, AL 35294, USA

**Keywords:** melanoma, tertiary lymphoid structures, immunotherapy, immune checkpoint inhibitors, acral melanoma, desmoplastic melanoma, uveal melanoma

## Abstract

Tertiary lymphoid structures (TLSs) are ectopic lymphoid aggregates often found in chronic inflammatory conditions, including cancer. These structures, which share many cellular and functional features with secondary lymphoid organs, can profoundly influence the tumor microenvironment by promoting local anti-tumor immune activation. TLSs have been observed in various cancers, including melanoma, and are associated with improved responses to immunotherapy and clinical outcomes. However, our understanding of the molecular mechanisms underlying TLS formation and function remains incomplete. This review summarizes the current findings on TLSs in human melanoma, drawing from multiple studies to provide an updated overview. We discuss the cellular composition, spatial distribution, and genetic signatures of TLSs at different stages of melanoma pathogenesis and in subtypes including acral, uveal, and desmoplastic melanoma. Additionally, we examine the influence of tumor mutational burden (TMB) and complement activation on TLS formation, as well as the role of TLSs in immune checkpoint inhibitor therapy. We also highlight the potential of TLSs as indicators for disease progression and treatment response, and review preclinical strategies aimed at inducing TLSs to improve therapeutic outcomes. This synthesis aims to support ongoing research into the role of TLSs in melanoma immunobiology.

## 1. Introduction

Tertiary lymphoid structures (TLSs) are ectopic lymphoid aggregates that form outside traditional lymphoid tissues such as the bone marrow, thymus, lymph nodes, and spleen. Initially identified within settings of chronic inflammation such as transplant rejection [1] and autoimmune disease [2], these structures exist along a spectrum, ranging from clusters of lymphocytes to highly organized formations with distinct T and B cell compartments, stromal cells, and high endothelial-like vessels expressing peripheral node addressin (PNAd). In their more complex form, TLSs closely resemble secondary lymphoid organs. The presence and function of TLSs have garnered significant attention due to their potential implications in tumor pathophysiology, particularly in how they shape the immune microenvironment and influence responses to immune-based therapies.

TLSs have been observed across numerous malignancies, including non-small cell lung cancer [3], colorectal carcinoma [4], breast cancer [5], ovarian cancer [6], squamous cell carcinoma of the head and neck [7], sarcoma [8], and melanoma [9], often correlating with enhanced responses to immunotherapy. Their presence is frequently associated with high densities of tumor-infiltrating T lymphocytes and improved patient survival, suggesting that TLSs may play a crucial role in enhancing anti-tumor immune responses by providing specialized environments for antigen presentation and activating cytotoxic CD8^+^ T cells. However, it is becoming increasingly clear that TLSs are not a uniform feature across tumor types, as they exhibit considerable variability in their cellular composition, location, and density.

Melanoma, a classically immunogenic cancer, continues to be a major global health concern. Melanoma has a multifactorial etiology that includes both ultraviolet (UV)-dependent and UV-independent pathways. Notably, melanoma can develop in non-sun-exposed areas, highlighting the importance of UV-independent mechanisms [10]. Key risk factors include UV radiation exposure, a history of blistering sunburns, immunosuppression, genetic predisposition, and the presence of changing moles [11,12]. Several genes have been studied in association with hereditary melanoma conditions including *CDKN2A*, BRACA1-associated protein 1 (*BAP1*), and *CDK4* [13].

While immunotherapies, such as programmed cell death protein 1 (PD-1) checkpoint inhibitors that improve anti-tumor immune responses, have revolutionized melanoma treatment [14], the disease remains responsible for over fifty thousand deaths annually, with incidence and mortality rates rising worldwide [15,16]. Projections indicate a dramatic increase in melanoma cases and deaths by 2040 [16]. Despite advancements, many patients still experience recurrence or disease progression [17], underscoring the need for a comprehensive understanding of immune responses in melanoma pathogenesis and therapy. While studies from other cancers have provided valuable insights into the role of TLSs, it is increasingly clear that TLS function is highly context-dependent, and their impact in melanoma requires focused exploration.

This review aims to synthesize current knowledge regarding TLSs in melanoma, with a particular emphasis on human cutaneous melanoma. We will examine what is known about the composition and function of TLSs at various stages of disease progression, while also highlighting unique features of acral, uveal, and desmoplastic melanoma variants. Additionally, we will explore the influence of TLSs on immune checkpoint inhibitor therapy in human melanoma, discussing how these structures may modulate treatment responses. Finally, we will review preclinical studies and proof-of-principle research on the therapeutic potential of inducing TLSs to enhance anti-tumor immunity in melanoma.

## 2. Cellular Classification and Composition of TLSs in Melanoma

**Figure 1:**
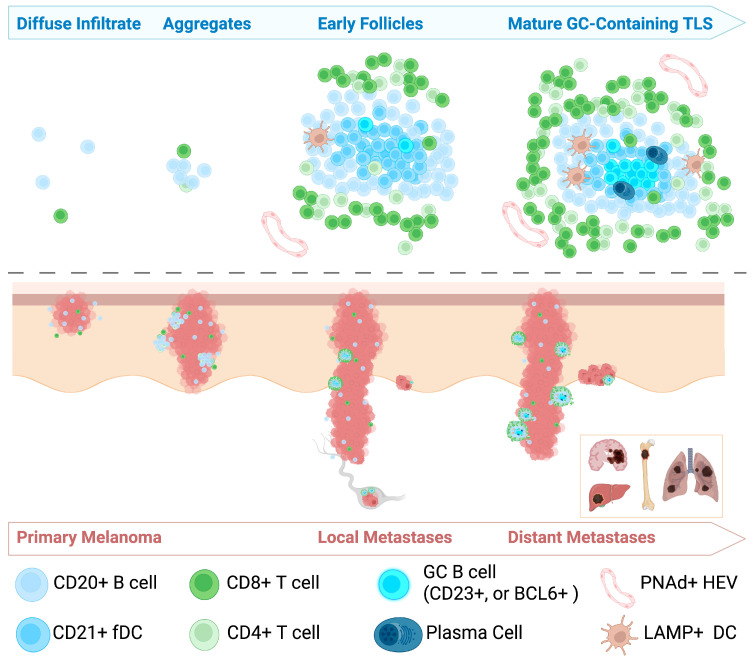
**Schematic representation of tertiary lymphoid structure (TLS) development during melanoma progression**. This cartoon illustrates the progression of TLS formation across stages of melanoma progression. The top half of the figure illustrates the evolution of TLS formation, progressing from diffuse immune cell infiltrates to small aggregates, early follicles, and mature germinal center (GC)-containing TLSs. The bottom half depicts melanoma progression from early primary tumors to deeper invasion, local lymph node involvement, and distant metastases to organs such as the brain, liver, bone, and lungs.

The immune composition and spatial organization of the tumor microenvironment (TME) in melanoma is both heterogeneous and dynamic. Melanoma is widely recognized as an immunologically “hot” tumor, characterized by robust immune cell infiltration [18]. Among these infiltrates, TLSs have been observed in a subset of melanomas. However, the definition and classification of TLSs in melanoma remain inconsistent. Some studies describe them broadly as lymphoid aggregates, while others require the presence of germinal centers (GCs) for formal classification. Several groups have attempted to standardize TLS identification methodologies [19,20,21]. For instance, Werner et al. [19] categorizes TLSs into early, primary follicular, and secondary follicular phenotypes with or without BCL6^+^ GCs. In their framework, primary follicular TLSs are identified by a central cluster of CD21^+^ follicular dendritic cells (FDCs), while the addition of CD23^+^ FDCs indicates a more mature, secondary follicular phenotype. In contrast, Karapetyan et al. classify TLSs as aggregates of more than 50 T or B lymphocytes, arranged in a concentric pattern and containing GCs—closely resembling Werner’s BCL6^+^ secondary follicles [22]. Despite differences in terminology and criteria, these studies converge on the understanding that the organization of lymphocytic infiltrates in melanoma evolves over time. This progression—from diffuse infiltration to increasingly structured aggregates—parallels tumor advancement and reflects the dynamic nature of the immune response in melanoma (Figure 1).

The lymphocytic infiltrate in primary human cutaneous melanoma is primarily composed of diffuse lymphocytes, with CD20^+^ B and CD8^+^ T lymphocytes being the most prevalent. In some human melanoma samples, the presence of PNAd^+^ high endothelial venules (HEV)-like vessels has been observed, suggesting a pathway for immune cells to traffic into the tumor tissue [9]. Primary melanoma typically lacks highly organized or complex TLSs, but several studies have identified early forms of lymphocytic organization into clusters and nodules [22]. These structures can consist of dense aggregates of CD20^+^ B cells interspersed with CXCL13-secreting cells and CD4^+^ T helper cells [19]. CXCL13, which binds its receptor CXCR5, is known to promote B cell trafficking and lymphoid organ formation [23]. In primary melanoma samples with a Breslow depth greater than 2 mm, these structures have been shown to be within 1 mm of the invasive tumor edge, suggesting a relationship between TLS formation and tumor progression [19]. Notably, primary melanoma lesions have been shown to lack significant *CD21L* gene expression, a marker indicative of FDCs, further suggesting the absence of well-organized lymphoid structures in these lesions [9]. Currently, the literature lacks sufficient data on the character and density of TLSs in radial versus vertical growth phase melanomas, though such comparisons could yield important insights in future studies.

In metastatic cutaneous melanomas, TLSs are present at higher densities and occupy a larger relative area than in primary cutaneous melanoma [9,19]. TLSs in metastatic melanoma were first identified by Cipponi et al. as aggregates of CD20^+^ B and CD8^+^ T cells that resembled follicles seen in control lymphoid tissue [9]. In this study, CD138^+^ plasma cells, CD21^+^ FDCs, and LAMP^+^ mature DCs were identified with heterogeneity between samples [9]. TLSs in metastatic melanoma have also demonstrated presence of CD86^+^ antigen-presenting cells [24]. FoxP3^+^ regulatory T cells (Tregs) have been identified within TLSs by various groups, but they are generally present in low numbers [24,25]. TLSs in metastatic melanoma have been observed in both intra- and extratumoral locations, with extratumoral sites being more common. As in primary melanomas, the highest density of TLSs in melanoma metastases occurs near the tumor perimeter [19,22,24].

Stratification of TLS subtypes has shown that early TLSs are the most common, followed by secondary follicular TLS-lacking GCs. In contrast, secondary follicular TLSs containing GCs—whether defined by BCL6 expression or the presence of activation-induced cytidine deaminase (AID) B cells—represent the rarest subtype [9,19]. The cellular composition of GCs in cutaneous melanoma metastases resembles that of control tonsillar tissue in that BCL6^+^CD20^+^ B cells and BCL6^+^CD4^+^ T cells are embedded within a network of CD21^+^CD23^+^ FDCs. Unlike physiological GCs, however, they lack classical polarity and exhibit limited proliferative activity, as indicated by reduced Ki67 staining [19]. Notably, subsequent studies have successfully identified highly proliferative B cell subsets within TLS of metastatic melanoma [26], but much remains to be understood about the underlying mechanisms dictating TLS composition and function in melanoma.

TLS composition also varies by metastatic site. BCL6^−^ secondary follicular TLSs are more abundant in lymph node metastases than in distant cutaneous lesions [19]. Visceral metastases to organs such as the lung, muscle, and gastrointestinal tract express *CD21L*, indicating the presence of mature FDCs and more organized TLSs. In contrast, liver and brain metastases lack *CD21L* expression, suggesting impaired TLS maturation at these sites [9].

An important caveat to note when assessing the heterogeneous character and spatial distribution of lymphoid structures between samples is the inherit variability in two-dimensional histologic assessment. Although existing studies have revealed information about general trends and progression of ectopic lymphoid maturation in melanomas, sectioning of tissue samples introduces sampling biases and variability that can affect quantitative and spatial assessments. Recent three-dimensional (3D) reconstructions of TLSs in colorectal cancer have shown that these structures are often large and multi-lobular, suggesting that evaluation across entire tissue volumes—rather than isolated 2D sections—may be necessary for accurately capturing TLS architecture and activity within tumors [27,28]. While similar 3D approaches have not yet been widely applied to melanoma, they may offer valuable insights in future investigations.

## 3. Molecular and Genetic Characters of TLSs in Melanoma

In addition to histological identification, emerging research has begun to uncover the molecular and transcriptional characteristics of TLSs in melanoma. Several studies have identified gene signatures that can be used to predict the presence of TLSs [24,26,29]. A 12-chemokine gene expression signature (GES)—originally developed to characterize immune structures in colorectal cancer—has proven predictive of TLS-like aggregates in melanoma as well. This signature includes *CCL2*, *CCL3*, *CCL4*, *CCL5*, *CCL8*, *CCL18*, *CCL19*, *CCL21*, *CXCL9*, *CXCL10*, *CXCL11*, and *CXCL13*, all of which are involved in immune cell recruitment and organization [4]. In stage IV melanoma metastases, high GES scores correlated with increased lymphocytic infiltration and more frequent detection of TLSs, particularly in peritumoral regions. While the relationship between GES and TLS density is not strictly linear, it highlights the potential predictive value of examining TLS-associated genetic expression signatures to identify TLS-rich tumors and aiding in the stratification of patient samples according to their immune landscape [24].

Immunoglobulin repertoire analysis of microdissected CD20^+^ B cell follicles from human cutaneous melanoma has provided evidence of a localized, antigen-driven B cell response, characterized by clonal expansion, class switch recombination, and somatic hypermutation—including IgA1 and IgA2 isotypes, despite the non-mucosal origin of these tumors [9]. Bulk RNA sequencing of melanoma samples with histologically confirmed lymphoid aggregates further supports these findings. These tumors demonstrate upregulation of MHC class II molecules, interferon response genes, IL-6 and STAT3 signaling, and cytokines that promote B cell differentiation (e.g., CCL19, CXCL13, TNFRSF17), while genes involved in pigmentation, synaptic signaling, and neurotransmitter pathways are downregulated [22]. Though the biological significance of this shift remains under investigation, it raises intriguing questions about the relationship between TLS, immune signaling, and melanoma differentiation states.

## 4. Impact of Tumor Mutational Status on TLS Formation

The propensity of metastatic melanoma to contain more mature TLSs than primary melanomas raises important questions about the relationship between tumor genetics and the development of these immune structures. Compared to primary tumors, metastatic melanomas typically exhibit a higher tumor mutational burden (TMB), often exhibiting mutations in genes such as *BRAF*, *NRAS*, and *PTEN* [30]. Investigation of cutaneous melanoma cohorts within The Cancer Genome Atlas (TCGA) database has revealed an inverse correlation between the expression of TLS-associated genes and those involved in key DNA repair pathways, including mismatch repair (MMR), non-homologous end joining (NHEJ), and homologous recombination (HR) [31]. This suggests that tumors with elevated mutational burden—often resulting from compromised DNA repair mechanisms—may be more likely to support TLS formation. However, the relationship between tumor mutational status and TLS development remains incompletely understood and likely varies across contexts. For example, preliminary evidence suggests that in the setting of anti-cytotoxic T-lymphocyte associated protein 4 (CTLA-4) or anti-PD-1 therapy, TLS gene expression may occur independently of the tumor’s mutational status [26]. Yet studies in other tumor types support the idea that specific oncogenic mutations may influence both the formation and function of TLS (reviewed in [31]). Nevertheless, these findings suggest a potential link between genomic instability and the recruitment or organization of local immune responses, positioning TMB not only as a possible predictor of TLS presence in treatment-naïve melanoma but also as a factor that may influence the functional maturity or impact of these structures.

## 5. Impact of Complement on TLS Formation

Emerging evidence has identified complement-mediated signaling as a significant regulator in the development of TLSs. A study by Zhang et al. investigated the relationship between complement and TLS emergence in melanoma. The formation of TLSs was detected by the expression of TLS-associated chemokines genes, including *CXCL12*, *CXCL13*, *CCL19*, *CXCR5*, and *LAMP3* [32]. Prior literature has characterized these molecules as key modulators in B cell and CD4^+^ and CD8^+^ T cell recruitment and organization, ultimately resulting in lymphoid neogenesis [26]. The authors of this study reported an increased level of all proteins, indicating a positive correlation between complement C2 and development of TLSs [32]. While these findings suggest that C2 may play a role in enhancing TLS density, which could contribute to improved immune responses within the TME and better responses to immunotherapy, it is important to note that this remains speculative. The underlying mechanisms are not yet fully understood, and no causal relationship has been established. Given the limited exploration of complement signaling in melanoma, further studies are warranted to investigate this potential association.

## 6. TLSs as a Biomarker for Melanoma

Given the apparent progressive development and maturation of TLS from simple lymphocyte clusters to fully functional, GC-containing structures in primary and metastatic melanoma, TLSs have been proposed as potential biomarkers for disease progression. Lynch et al. investigated this concept in a study of patients with melanoma who were naïve to immune or chemotherapy treatments, examining tumor samples from partial or complete resections. Increased amounts of B cells and CD8^+^ T cells were detected within TLSs, potentially contributing to the observed overall survival also reported in patients with these lymphoid structures. More specifically, this group reported longer survival in patients with a smaller percentage of CD21^+^ B cells, which are normally theorized to result in immune suppression within the TME [33]. On the contrary, higher amounts of AID^+^ B cells were associated with improved survival outcomes, which are likely rooted in the immune-mediated control of tumor progression via the AID within the existing B cells. Variation in the constituents of TLSs is an essential component modulating the TME and clinical outcomes as indicated by this study [33]. The association between TLSs and responses to immunotherapies in melanoma—some of which will be discussed below—has been explored in numerous studies. The potential utility of TLSs as a prognostic indicator for treatment response has been recently discussed by Padonou et al. [34].

## 7. TLSs and Immune Checkpoint Inhibitor Therapy in Melanoma

The influence of TLSs on immunotherapy is highly context-dependent and closely linked to their cellular composition [33]. Immunotherapies aim to enhance the immune response against cancer, often by blocking inhibitory pathways that tumors use to evade immune detection or by augmenting pathways that promote anti-tumor responses. Immunotherapies, such as PD-1 and PD-L1 inhibitors, as well as anti-CTLA-4 agents, have shown varying levels of efficacy depending on the presence and characteristics of TLSs within the TME. As summarized below and in Table 1, recent studies have provided more insight into how the cellular makeup of TLSs can modulate the response to these therapies, shedding light on their potential role in enhancing or hindering treatment success.

### 7.1. Anti-PD-L1 and Anti-PD-1

A study conducted by Zhang et al. investigated the influence of nivolumab, an anti-PD-1 monoclonal antibody commonly utilized for melanoma treatment, on the presence of C2 levels, survival times, and TLS formation. They found an increase in C2 levels in the treatment responsive group, highlighting activation of the complement activation system within the TME. Individuals with higher C2 levels had an improved clinical prognosis with greater survival times. This was proposed to be due to complement-mediated cytotoxicity against tumor cells as C2 levels rose post-anti-PD-1 therapy. Furthermore, higher C2 levels were associated with decreased ulceration, a process typically associated with increased mitotic activity and tumor thickness [32]. The findings from this study suggest both C2 and TLSs to be protective factors with implication of favorable outcomes in melanoma and potentiators of immunotherapy response.

Helmink et al. similarly pursued the functional impact of TLSs in melanoma after administration of anti-PD-1 therapy. The findings from this study revealed the presence of B cells within lymphoid structures. Furthermore, increased expression of B cell-associated genes in TLSs were associated with higher response to anti-PD-1 therapy. Transcriptomic analyses and mass cytometry revealed differential expression of B cell markers in responders compared to non-responders. Tumors of responders also exhibited switched memory B cells, B cell clonal expansion, and B cells with unique functional states that may underlie the treatment response seen. Of note, this study explored the use of PD-1 inhibitors in the presence and absence of CTLA-4 inhibitors. The previously mentioned findings were independent of the simultaneous treatment with CTLA-4 inhibitors, indicating that the sole blockade of PD-1 may be sufficient to enhance immune function and for treatment of melanoma [25].

Similar findings were obtained by Cabrita et al. in which patients receiving anti-PD-1 treatment had higher levels of TLSs, increased survival time, and exhibited a strong treatment response. Furthermore, this study revealed that presence of TLSs within the TME is a positive indicator of responsiveness to PD-1 inhibitors regardless of the melanoma mutational load [26]. Reinforcing previous findings, B cells significantly correlated with TLS presence in addition to T cells and various other immune cells [26]. As indicated earlier, TLSs are key mediators of an immune-mediated response produced in a given tumor TME. Thus, upregulation of TLS formation and activity remains a key focus in the development of melanoma therapy for optimization of clinical response to immune-mediated therapies.

### 7.2. Anti-CTLA-4

Stratifying melanomas by TLS gene expression signature revealed that tumors with high TLS levels were associated with significantly increased survival following CTLA-4 blockade [26]. Additionally, pretreatment with CTLA-4 inhibitors prior to anti-PD-1 therapy has been linked to better overall survival compared to monotherapy, anti-PD-1 pretreatment, or concurrent administration [38]. This therapeutic benefit may, in part, be influenced by the activity of immunosuppressive T cell subsets within the TME, particularly Tregs and follicular regulatory T cells (Tfrs).

Both Treg and Tfr cells express high levels of PD-1, but Tfrs can be further distinguished by expression of BCL6 and CXCR5, as well as their localization within lymph node GCs where they suppress B cell and T follicular helper activity [39,40]. Tfrs have not been extensively studied or histologically validated within TLSs of human melanoma; however, metanalyses of single-cell RNA sequencing from three human melanoma datasets [41,42,43] suggests that Tfr-like populations are present [38]. In murine B16F10-OVA tumor models, these cells have been shown to increase over time and exhibit immunosuppressive functions. It is postulated that anti-CTLA-4 treatment depletes or impairs the immunosuppressive activity of Tfrs, thereby allowing for more effective anti-PD-1-mediated immune activation and treatment response [38].

## 8. Select Variants

### 8.1. Acral Melanoma

While extensive research has been conducted on the role of TLSs in cutaneous melanoma, limited studies have explored TLSs in the setting of acral melanoma (AM). These tumors are rare in occurrence and associated with a lower tumor mutational burden when compared to other cutaneous counterparts [37]. AMs arise on distal parts of the extremities and are independent of exposure to ultraviolet radiation. Mo et al. highlighted that the amount of TLS infiltration had no correlation with survival outcome in AM post PD-L1 inhibition. Though further analysis revealed higher amounts of CD4^+^ T cells in groups with higher TLS levels, this contrasted with the UV-induced melanoma group that demonstrated a robust increase in CD4^+^ T cells, CD8^+^ T cells, B cells, and dendritic cells in cell-rich TLSs [44]. This finding suggests that utilizing TLSs as a therapeutic target and a prognostic indicator may be limited in AM. On the contrary, the work of Su et al. found intratumoral TLSs to consist of increased numbers of CD4^+^ and CD8^+^ T cells whereas surrounding regions had an increased presence of CD68^+^ macrophages. A relationship between CD8^+^ T cells and CD68^+^ macrophages was also evident as the presence of both cell types is key in modulating immune response in the TME.. Furthermore, this study revealed longer survival rates for patients with TLSs, especially those located within the TME [37].

### 8.2. Uveal Melanoma

Uveal melanoma (UM) is another rare form of melanoma with limited studies on potential therapeutic options. The findings of a small, 29-person clinical trial by Sah et al. support the plausibility of targeting CCL21 via immunotherapy given the increased presence of CCL21 in TLS-positive samples of UM and its association with longer progression-free and overall survival. Additionally, pretreatment plasma levels of CCL21 were higher in patients who exhibited a partial response than those who exhibited progressive disease while undergoing immunotherapy. Furthermore, CCL21 was implicated in increasing expression of naïve T cells and dendritic cells in the TME and B cell activation, resulting in organization of TLSs and potentiation of anti-tumor immunity [36].

### 8.3. Desmoplastic Melanoma

Desmoplastic melanoma (DM) is a subtype of melanoma typically arising in sun-damaged skin of elderly individuals and is characterized by a high TMB [45]. This subtype demonstrates enhanced responsiveness to PD-1 blockade [35], a phenomenon that may be linked to its unique TLS composition. While TLSs in primary DM resemble those described in non-desmoplastic cutaneous melanomas, small differences may contribute to their unique pathophysiology and treatment response. TLSs in primary DM consist of a CD20^+^ B cell core, surrounded by CD8^+^ T cells, CD83^+^ dendritic cells, and PNAd^+^ vasculature, with scattered FoxP3^+^ Tregs throughout [46]. While TLSs in non-desmoplastic melanoma tend to localize peritumorally, TLSs in “pure” primary DM are predominantly intratumoral and show higher densities of proliferative CD8^+^ T cells and CD20^+^ B cells [47].

Given the partially controversial and limited findings from the aforementioned studies, further investigation exploring the molecular mechanisms of TLSs in various forms of melanoma is warranted. Future works should explore the feasibility of utilizing TLSs as prognostic indicators or even therapeutic targets. Characterizing TLSs with respect to tumor genotype and overall immune cell composition may also be beneficial to the improvement of melanoma immunotherapy strategies.

## 9. Preclinical Strategies for Inducing Tertiary Lymphoid Structures in Immunotherapy

As our understanding of TLS development, function, and clinical relevance deepens, there is growing interest in therapeutically manipulating these structures for prophylactic or therapeutic benefit. Since not all human tumors naturally form TLS, strategies to induce them may offer a novel means of enhancing response to immunotherapy.

Zhu et al. developed a preclinical murine model in which subcutaneous implantation of lymph node-derived stromal cells expressing a robust 12-chemokine TLS gene signature successfully induced TLSs in vivo. These induced TLSs activated lymphocytes, increased IFN-γ production, and promoted anti-tumor T cell responses, notably inhibiting the growth of subsequently injected melanoma cells [48]. However, their ability to control pre-existing tumors remains uncertain and likely depends on the timing and kinetics of TLS induction.

Intratumoral administration of stimulator of interferon genes (STING) agonists has also been explored as a strategy to promote TLS formation in melanoma. In a murine B16.F10 model, intralesional injection of a pharmacologic STING agonist led to upregulation of TLS-associated chemokines and cytokines within the tumor microenvironment, along with enhanced dendritic cell maturation. However, the lymphoid aggregates induced by STING activation lacked robust B cell infiltration and germinal center formation characteristic of mature TLS in human metastatic melanoma [49]. Similarly, intralesional IL-15-expressing oncolytic adenovirus has been shown to induce formation of nonclassical, premature TLS-structures within in B16.F10 tumors in a STING-dependent manner [50]. These findings suggest that while STING pathway activation can initiate TLS-like structures, additional signals—such as those promoting B cell recruitment and TLS maturation—may be required to generate fully functional TLSs and maximize therapeutic benefit.

Yoshimitsu et al. successfully induced B cell-rich TLSs in a murine melanoma model using a combinatorial chemokine strategy. In mice with established subcutaneous B16.F0 tumors, peritumoral injection of CXCL13 and CCL21, combined with intraperitoneal anti–PD-L1 therapy, increased intratumoral TLS formation and significantly enhanced tumor suppression [51]. Similarly, TLS-like structures have been induced using lymphotoxin-α (LTα), a potent driver of lymphoid genesis [52,53,54]. A tumor-specific antibody-LTα fusion protein has been shown to exhibit cytotoxic activity against syngeneic murine melanoma, accompanied by functional lymphoid organization comprising B and T cell aggregates, MHC class II^+^ antigen-presenting cells, L-selectin^+^ T cells, and PNAd^+^ high endothelial venules [55].

## 10. Conclusions

In conclusion, TLSs are a dynamic and integral component of the melanoma tumor immune microenvironment. Although challenges remain in accurately characterizing their composition, distribution, and function, it is clear that TLSs significantly influence melanoma pathophysiology and treatment outcomes. TLSs evolve from diffuse lymphocytic infiltrates in primary tumors to more organized structures in metastatic disease, with their formation influenced by factors such as TMB, complement pathways, and melanoma subtype.

There is strong support that TLSs are strongly associated with improved responses to immunotherapies, potentially due to their ability to serve as specialized local hubs of immune activation to enhance anti-tumor immunity. TLSs have demonstrated potential as biomarkers for disease progression and treatment response, offering promise for both prognostic evaluation and therapeutic stratification. Preclinical studies have begun exploring ways to induce TLSs and harness their therapeutic potential. As we deepen our understanding of the complex role and regulation of TLSs, we will be better equipped to fully harness their clinical potential, ultimately improving personalized treatment strategies and patient outcomes in melanoma.

## Figures and Tables

**Table 1:** Utility of TLSs in predicting a therapeutic response in treatment of various human melanoma subtypes.

Treatment(s)	Association with TLS	References
**Anti-PD-1**	Increased presence of TLSs may improve treatment response of anti-PD-1 in melanoma and leads to favorable survival outcomes Cutaneous melanoma Acral melanoma Uveal melanoma Desmoplastic melanoma	[25,32,35,36,37]
**Anti-PD-1 and Anti-CTLA4**	Tumor TLS gene signatures predict overall survival in patients with metastatic melanoma treated with anti-PD-1 and anti-CTLA4 Cutaneous melanoma	[26]
**Anti-CTLA4 prior to Anti-PD-1**	Treatment with anti-CTLA4 prior to anti-PD-1 reduces Tfr activity and increases tumor control and survival at higher rates compared to sole usage of anti-PD-1 and other treatment sequences Cutaneous melanoma	[38]

## Data Availability

No new data were created or analyzed in this study.
